# SOX4 interacts with EZH2 and HDAC3 to suppress microRNA-31 in invasive esophageal cancer cells

**DOI:** 10.1186/s12943-014-0284-y

**Published:** 2015-02-03

**Authors:** Rainelli B Koumangoye, Thomas Andl, Kenneth J Taubenslag, Steven T Zilberman, Chase J Taylor, Holli A Loomans, Claudia D Andl

**Affiliations:** Department of Surgery, 2213 Garland Ave. 10445 MRB IV, Nashville, TN 37232-6840 USA; Division of Dermatology, Department of Medicine, 21st Ave South, A-2310 Medical Center North, Nashville, TN 37232-6840 USA; Department of Cancer Biology, 2213 Garland Ave. 10445 MRB IV, Nashville, TN 37232-6840 USA; Vanderbilt Ingram Cancer Center, Vanderbilt University Medical Center, Nashville, TN 37232-6840 USA; Vanderbilt Digestive Disease Center, Vanderbilt University Medical Center, Nashville, TN 37232-6840 USA

**Keywords:** Esophageal cancer, Oncogene, MicroRNA, miR-31, EZH2, SOX4, HDAC3, Epigenetics

## Abstract

**Background:**

Tumor metastasis is responsible for 90% of cancer-related deaths. Recently, a strong link between microRNA dysregulation and human cancers has been established. However, the molecular mechanisms through which microRNAs regulate metastasis and cancer progression remain unclear.

**Methods:**

We analyzed the reciprocal expression regulation of miR-31 and SOX4 in esophageal squamous and adenocarcinoma cell lines by qRT-PCR and Western blotting using overexpression and shRNA knock-down approaches. Furthermore, methylation studies were used to assess epigenetic regulation of expression. Functionally, we determined the cellular consequences using migration and invasion assays, as well as proliferation assays. Immunoprecipitation and ChIP were used to identify complex formation of SOX4 and co-repressor components.

**Results:**

Here, we report that SOX4 promotes esophageal tumor cell proliferation and invasion by silencing miR-31 via activation and stabilization of a co-repressor complex with EZH2 and HDAC3. We demonstrate that miR-31 is significantly decreased in invasive esophageal cancer cells, while upregulation of miR-31 inhibits growth, migration and invasion of esophageal adenocarcinoma (EAC) and squamous cell carcinoma (ESCC) cell lines. miR-31, in turn, targets SOX4 for degradation by directly binding to its 3′-UTR. Additionally, miR-31 regulates EZH2 and HDAC3 indirectly. SOX4, EZH2 and HDAC3 levels inversely correlate with miR-31 expression in ESCC cell lines. Ectopic expression of miR-31 in ESCC and EAC cell lines leads to down regulation of SOX4, EZH2 and HDAC3. Conversely, pharmacologic and genetic inhibition of SOX4 and EZH2 restore miR-31 expression. We show that SOX4, EZH2 and HDAC3 form a co-repressor complex that binds to the miR-31 promoter, repressing miR-31 through an epigenetic mark by H3K27me3 and by histone acetylation. Clinically, when compared to normal adjacent tissues, esophageal tumor samples show upregulation of SOX4, EZH2, and HDAC3, and EZH2 expression is significantly increased in metastatic ESCC tissues.

**Conclusions:**

Thus, we identified a novel molecular mechanism by which the SOX4, EZH2 and miR-31 circuit promotes tumor progression and potential therapeutic targets for invasive esophageal carcinomas.

## Background

MicroRNAs (miRNAs) are a class of highly-conserved, noncoding 18-25- nucleotide RNAs that function as negative regulators of gene expression at the post-transcription level, binding to the 3′-untranslated region (3′-UTR) of mRNAs transcripts and targeting them for degradation [1]. Though implicated in carcinogenesis, it is not clear how miRNAs promote tumorigenesis and metastasis or what networks regulate miRNAs expression. miRNA expression is commonly dysregulated in human cancers, [2,3] including esophageal cancers [4].

miR-31 expression is altered in multiple human cancers. Depending on the cellular context, miR-31 may be up- or downregulated, acting as an oncogene or tumor suppressor, respectively. Overexpression of miR-31 has been linked to disease progression in colorectal cancer [5], head-and-neck squamous cell carcinoma (HNSCC) [6] and lung cancer [7]. miR-31 is downregulated in certain T-cell leukemias [8], breast cancer [9,10], melanoma [11], ovarian cancer [12] and prostate cancer [13]. Downregulation and loss of miR-31 in esophageal adenocarcinoma (EAC) correlates with poor patient prognosis [14-16]. Additionally, miR-31 expression is reduced in EAC patients with poor histomorphologic response to neoadjuvant chemoradiation therapy [17]. Conversely, miR-31 is upregulated in serum and tissue samples of esophageal squamous cell carcinoma (ESCC), with expression correlating to staging [18]. Yet, in another ESCC cohort miR-31 expression was decreased, and low miR-31 expression correlated with poorly differentiated tumors and decreased survival [19]. These reports emphasize the complexity of miR-31-associated phenotypes and the need to better define miR-31 targets, as well as pathways regulating miR-31 expression in different cancers.

SOX4 is a member of the highly conserved SoxC (SRY-related high-motility group box) transcription factors family, which contains two other members, SOX11 and SOX12 [20]. SOX4 is a putative stem cell marker that plays a crucial role during cell fate determination [21,22]. SOX4-deficient mice suffer from multiple developmental defects, dying at embryonic day 14, secondary to ventricular outflow tract malformation [23]. During embryogenesis, SoxC members are highly expressed, helping to maintain survival of pluripotent mesenchymal and neural progenitor cells [24]. In adults, expression of SOX4 is restricted to certain cell types, including hematopoietic stem cells, mammary stem cells and hair follicle stem cells [25-27].

Meta-analysis has identified SOX4 as one of the 64 genes that constitute a general signature in all human cancers, and genome wide promoter analysis has shown that SOX4 regulates the transcription of genes involved in TGF-β, Wnt, Hedgehog, and Notch pathways and components of miRNA processing machinery such as Dicer, Argonaute 1 and RNA Helicase A [28,29]. SOX4 induces EMT and breast cancer progression by cooperating with oncogenic Ras. More recent work shows that SOX4 induces EMT via the polycomb epigenetic regulator EZH2 [30]. miRNAs, such as miR-335, are known to target SOX4, suppressing metastasis and migration in breast cancer [3].

Polycomb group proteins have been linked to tumor progression in many cancers. The polycomb proteins can form at least two complexes: polycomb-repressive complexes 1 and 2 (PRC1 and PRC2). PRC2 contains three core proteins, EZH2, SUZ12, and EED. The histone methyltransferase EZH2 (enhancer of zeste homolog 2) epigenetically regulates genes involved in cell fate determination. Specifically, EZH2 trimethylates nucleosomal histone H3 at lysine 27 (H3K27me3). The H3K27me3 mark is associated with gene silencing and often found in the promoter of developmental genes [31,32]. However, it is unclear how EZH2 is recruited to the promoters it targets. Recent studies have shown that EZH2 interacts with various transcription factors such as androgen receptor (AR), GATA4, RORα and STAT3 and may directly activate or repress these genes independent of the H3K27me3 mark or chromatin modification [33-36]. With respect to miRNAs, prior work demonstrates that EZH2 interacts with AR to silence miR-31 in prostate cancers, and C-MYC recruits EZH2 to the miR-29 promoter in B-cell lymphomas [37].

EZH2 is upregulated in multiple cancers, promoting invasion and metastasis [38-40]. Genetic and epigenetic loss of miR-31 is associated with EZH2 overexpression in melanoma [11], suggesting that miR-31 directly or indirectly regulates EZH2 expression. Interestingly, studies show that polycomb complexes silence the CDKN2A and CDKN2B loci, which encode the tumor suppressors p14 (ARF), p15 (INK4B) and p16 (INK4A) [41] and contain the MIR31HG locus on chromosome 9 [42]. In line with this observation, Yamagishi *et al.* reported that PRC2 binds the miR-31 coding region and directly represses transcription of miR-31 in adult T-cell leukemia [8]. SOX4 positively regulates EZH2, indicating a potential functional link between miR-31, EZH2 and SOX4.

The roles of SOX4, HDAC3 and EZH2 in microRNA regulation are largely unknown and have been poorly defined so far. In this study, we explore the role of SOX4 and EZH2 in miR-31 repression and the contribution of miR-31 to survival, migration and invasion of aggressive esophageal cancers cells. We identify SOX4 as a direct target of miR-31. Expression of miR-31 inhibits SOX4, EZH2 and HDAC3 expression. We show that miR-31 is repressed in invasive esophageal cancers cell lines and that miR-31 levels inversely correlate with SOX4, EZH2 and HDAC3 expression. Co-immunoprecipitation demonstrates that SOX4 interacts with EZH2 and that HDAC3 may be important to bridge this interaction. We show that EZH2 and HDAC3 bind to the miR-31 promoter using chromatin immunoprecipitation. Altogether, our results identify a feed-forward loop that leads to the activation of SOX4, which in turn up-regulates and binds to EZH2, cooperating with HDAC3 to repress the miR-31 promoter and advance esophageal tumorigenesis.

## Results

### miR-31 expression is downregulated in invasive esophageal cancer cells

**Figure 1:**
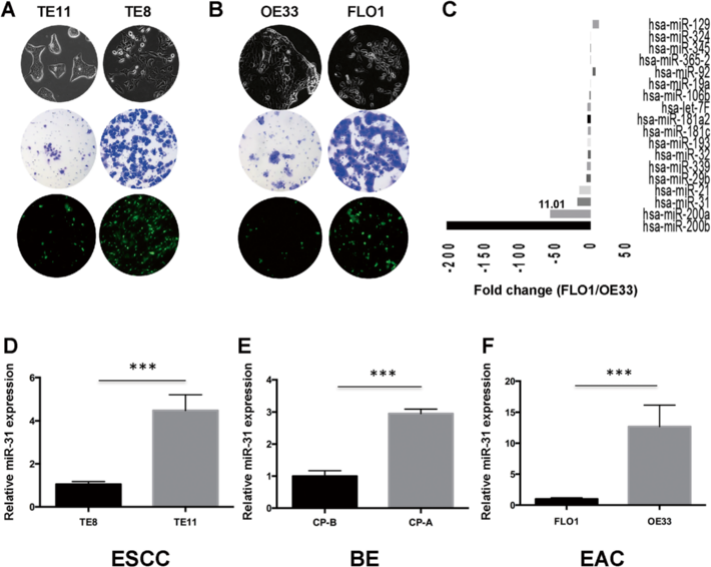
**miR-31 is downregulated in invasive esophageal cancer cells. (A, B)** Morphologic, migration and invasive capability of two ESCC cells lines and two EAC cell lines were analyzed by bright field microscopy and Boyden chamber transwell assays. **(C)** Fold change in expression of 18 miRNAs between the invasive EAC cell line FLO1 and non-invasive cell line OE33. **(D-F)** Quantitative RT-PCR for relative expression of miR-31. miR-31 expression was normalized to RNU6. **(D)** High expression of miR-31 in non-invasive ESCC TE11 cells versus invasive TE8 cells. **(E)** Higher expression in the CP-A cell line compared to CP-B and **(F)** non-invasive EAC OE33 compared with FLO1. Means ± SD from at least three biological replicates.

To investigate the role of miR-31 in esophageal cancers, we examined the expression of miR-31 in ESCC, EAC and Barrett’s esophagus cell lines of differing invasive potential (Figure 1). Comparing esophageal squamous cell carcinoma cell lines, TE11 is less motile than TE8 and displays an epithelial phenotype (Figure 1A). The esophageal adenocarcinoma cell lines OE33 and FLO1 differ in that FLO1 is more mesenchymal and therefore more motile than the OE33 (Figure 1B). After miR-200a and 200b, which are known for their roles in EMT, miR-31 was the most downregulated miRNA in invasive FLO1 cells compared to their less invasive OE33 counterparts by qPCR screen (Figure 1C). Similarly, miR-31 downregulation was observed in TE8 ESCC cell lines compared to TE11 (Figure 1D). Likewise, miR-31 expression was higher in non-invasive cell lines such as the benign Barrett’s esophagus cell line CP-A compared to the metaplastic CP-B cell line (Figure 1E). Furthermore, we confirmed the elevated miR-31 expression in OE33 cells, which have an epithelial phenotype, compared to FLO1 cells (Figure 1F). Next, to focus on the biological significance and regulatory mechanisms of miR-31 expression in invasive adenocarcinoma and squamous cell carcinoma, we expressed miR-31 in invasive ESCC and EAC cell lines and analyzed the effects on cell migration and invasion.

### miR-31 suppresses migration and invasion of aggressive ESCC and EAC cells

**Figure 2:**
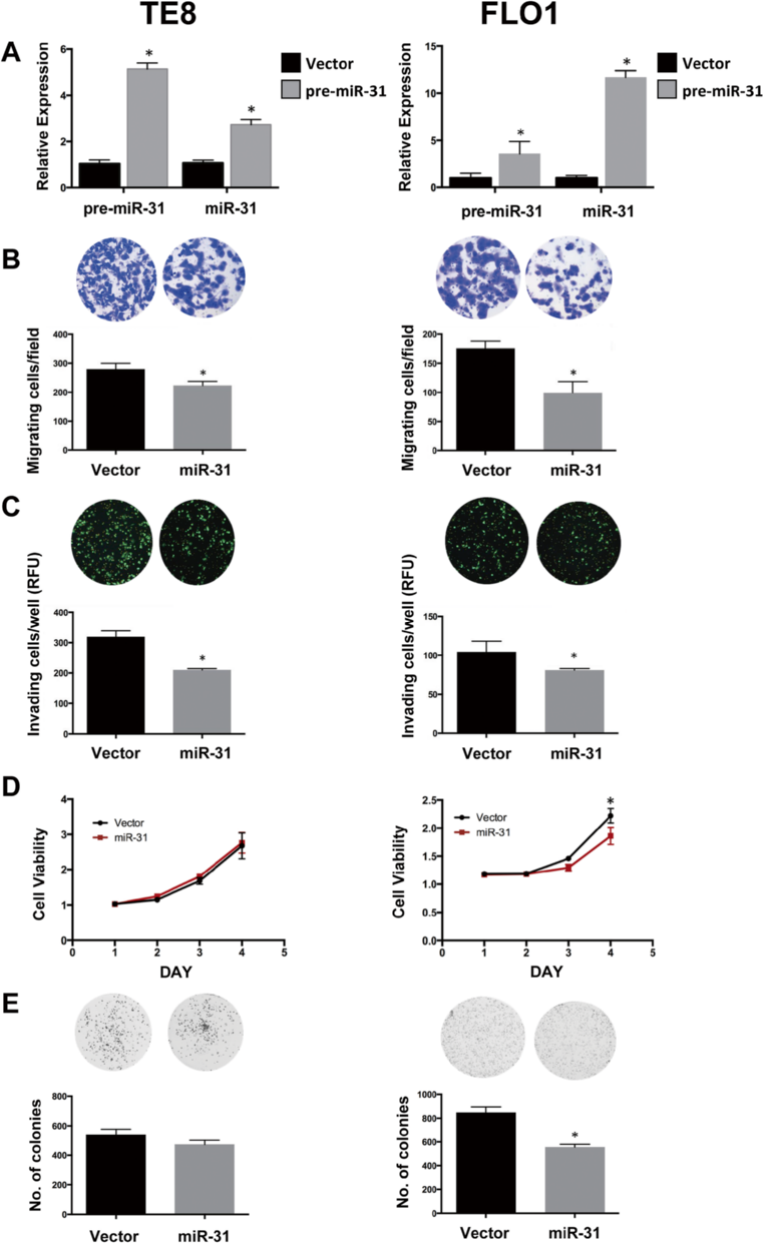
**Ectopic expression of miR-31 suppresses migration and invasion of ESCC and EAC cell lines.** TE8 and FLO1 cells were transfected with pre-miR-31 containing vector (grey bars) or empty vector control (black bars). **(A)** Overexpression of miR-31 was verified by qRT-PCR. Pre-mature miR-31 expression was normalized to GAPDH and mature miR-31 expression was normalized to RNU6. **(B)** Cell migration was measured 24 hours post-transfection using Boyden chambers. **(C)** Cell invasion was measured 24 hours post-transfection using Matrigel-coated Boyden chambers. **(D)** Cell viability was measured using the WST-1 assay. **(E)** Cell growth was evaluated by colony formation assay and measured on the Oxford Optronix Gelcount. Results are means ± SD from at least three biological replicates.

To examine the functional contribution of miR-31 in aggressive esophageal cancer, we transfected TE8 and FLO1 cells with vectors containing the precursor of miR-31 or an empty vector control. Ectopic expression of precursor and mature miR-31 in the respective cell lines was tested by quantitative RT-PCR (Figure 2A). In Boyden chamber migration and invasion assays, precursor miR-31 transfection significantly decreased cell migration and invasion in TE8 and FLO1 cells (Figure 2B, C, respectively). miR-31 expression had no significant effect on proliferation in TE8 cells and did not alter the number of colonies in colony formation assays (Figure 2D, E). In FLO1 cells, however, miR-31 suppressed proliferation and colony formation (Figure 2D, E, respectively), indicating miR-31 regulates esophageal carcinoma cell growth in some cell lines. These data suggest that miR-31 suppresses esophageal cancer cell motility and invasiveness, but cell growth depending on the cellular context.

### miR-31 is epigenetically repressed in invasive esophageal cancer cells

**Figure 3:**
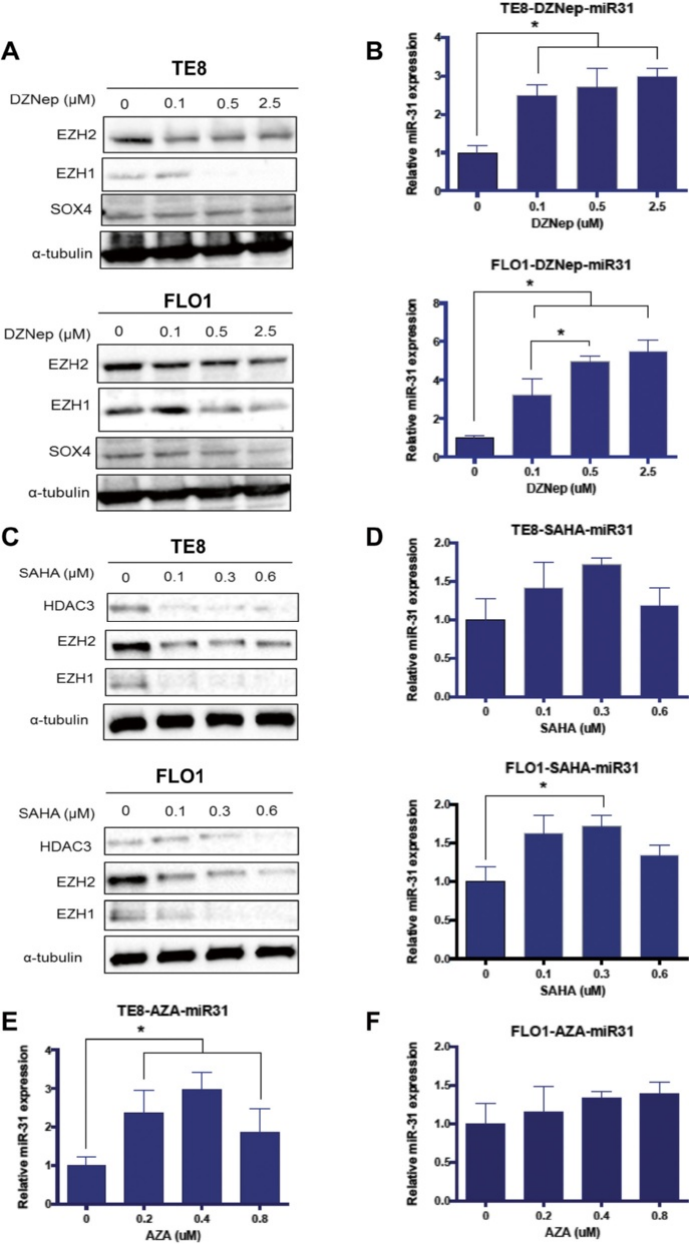
**miR-31 is epigenetically repressed in invasive esophageal cancer cells.** TE8 and FLO1 cells were treated at indicated concentrations with Polycomb/EZH2 inhibitor (DZNep), Histone deacetylase inhibitor (SAHA) and DNA methyl-transferase inhibitors (AZA). **(A)** DZNep treatment for 72 hours dose-dependently inhibits EZH2 and EZH1 in FLO1 and TE8 cells as shown by western blotting. **(B)** qRT-PCR analysis of miR-31 expression in TE8 and FLO1 cells treated with DZNep for 72 hours. **(C)** SAHA treatment for 48 hours inhibits HDAC3, EZH2 and EZH1 in TE8 and FLO1 cells in a dose-dependent manner. **(D)** qRT-PCR analysis of miR-31 expression in TE8 and FLO1 cells treated with SAHA for 48 hours. **(E-F)** qRT-PCR analysis of miR-31 expression in TE8 and FLO1 cells treated with 5-aza-deoxycytidine (AZA) for 72 hours. miR-31 expression was normalized to RNU6. Results are means ± SD from at least three biological replicates, ANOVA was used for statistical analysis.

Prior studies report that miR-31 expression is epigenetically silenced through promoter hypermethylation at CpG islands, as well as polycomb-mediated histone methylation [8,11]. We therefore speculated that loss of miR-31 in invasive esophageal cancer cells could be mediated, in part, by DNA and histone methylation. To determine the effect of PRC2 on miR-31 expression, we utilized the PRC2 inhibitor, 3-deazaneplanocin (DZNep). DZNep treatment of TE8 and FLO1 cells resulted in a decrease in EZH2 expression and caused a dose-dependent increase in miR-31 expression in both of our invasive cell lines (Figure 3A, B, respectively). DZNep treatment also led to a decrease in SOX4 protein level in FLO1 cells (Figure 3A). SOX4 was recently shown to upregulate EZH2 expression [30]. Using Western blot and qRT-PCR, we evaluated the effect of a pan-HDAC inhibitor (SAHA) on miR-31 expression. We found that SAHA led to a decrease in HDAC3, EZH2 and EZH1 protein levels in TE8 and FLO1 cells (Figure 3C). Most importantly, miR-31 expression was significantly upregulated in both cell lines following SAHA treatment (Figure 3D). To test whether promoter methylation at CpG islands was involved in miR-31 silencing, we used the DNA methylation inhibitor 5′AZA-Deoxy-Cytidine (AZA). Treatment with AZA significantly increased the expression of miR-31 in TE8 cells and to a lesser extent in FLO1 cells (Figure 3E and F). These data suggest that PRC2, HDAC and DNA methylation are involved in miR-31 epigenetic silencing.

### miR-31 directly targets SOX4 and indirectly targets EZH2 and HDAC3

**Figure 4:**
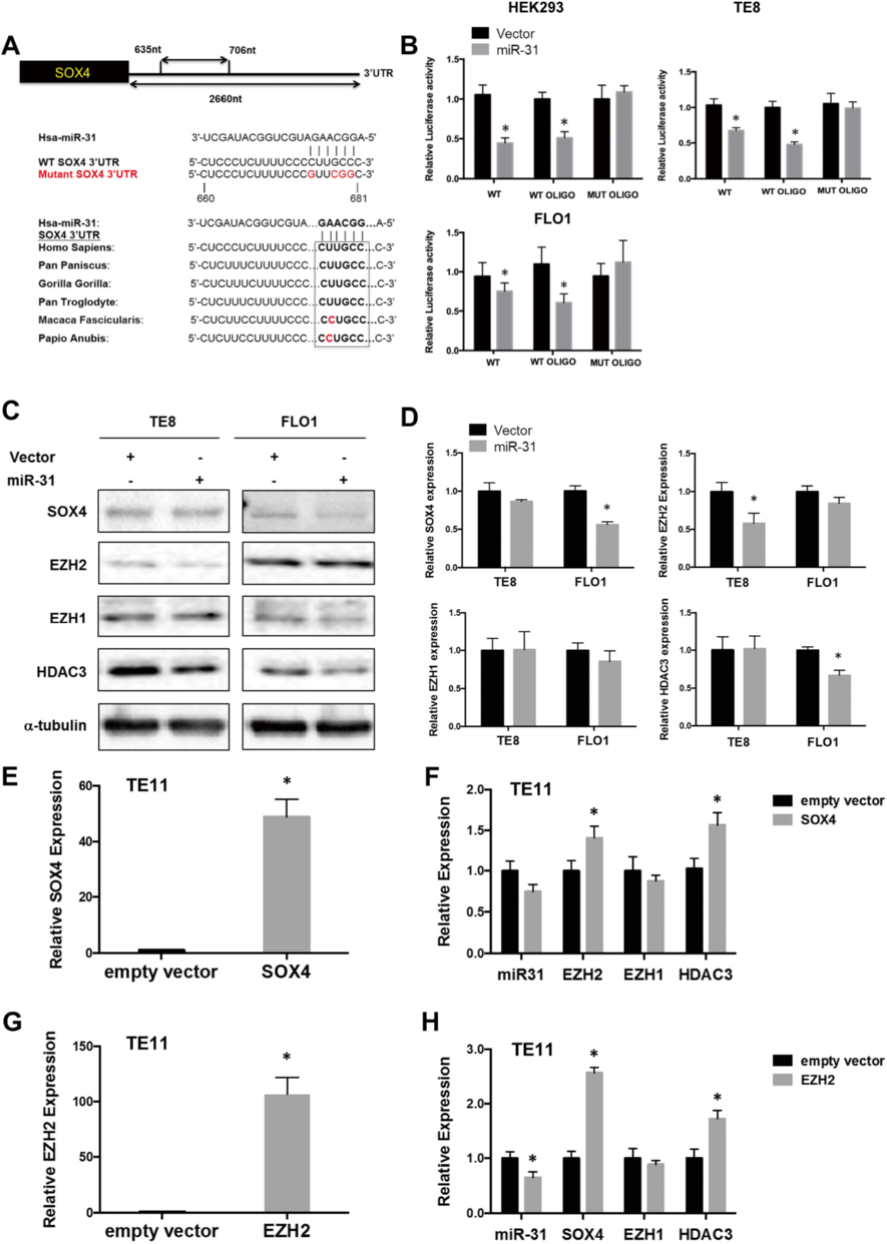
**miR-31 directly targets SOX4 and indirectly targets EZH2 and HDAC3. (A)** Computational analysis revealed one miR-31 binding site in the 3′UTR of SOX4. The upper panel shows the region containing the miR-31 binding site. The mutated SOX4 3′UTR seed region is indicated. A SOX4 3′UTR fragment containing wild type (WT OLIGO) or mutant (MUT OLIGO) of the miR-31-binding sequence was cloned into the downstream of the luciferase reporter gene. The lower panel shows the nucleotide sequence alignment of the predicted miR-31 binding site in the 3′UTR of SOX4 of six species. **(B)** HEK293, TE8 and FLO1 cells were co-transfected with psiCHECK-2 dual Renilla/Firefly luciferase plasmid containing either wild-type, wild-type oligo or mutant oligo of SOX4 3′UTR (indicated as WT, WT OLIGO and MUT OLIGO) with either pBABE empty vector control or pBABE-miR-31 vector. Luciferase activity was determined 48 hrs after transfection. **(C)** TE8 and FLO1 cells were transfected with miR-31 vector or empty vector control and cell lysates were analyzed after 72 hrs for SOX4, EZH2, EZH1 and HDAC3 by western blotting. α-tubulin was used as an internal control. **(D)** qRT-PCR analysis of SOX4, EZH2, EZH1 and HDAC expression in TE8 and FLO1 cells transfected with miR-31 or empty vector control. **(E, F)** qRT-PCR analysis of TE11 cells transfected with SOX4 or empty vector control. **(G, H)** qRT-PCR analysis of TE11 cells transfected with EZH2 or empty vector control. miR-31 expression was normalized to RNU6 and SOX4, EZH2, EZH1 and HDAC3 were normalized to GAPDH. Results are means ± SD from at least three biological replicates.

A study by Asangani *et al*. recently showed that genetic and epigenetic loss of miR-31 leads to a feed forward upregulation of EZH2 [11]. However, no mechanism was proposed. Previously, EZH2 was reported to interact with HDAC3 to repress miR-29 in lymphomas [37]. More recent work shows that SOX4 binds to the EZH2 promoter, thereby upregulating EZH2 expression [30]. We hypothesized that SOX4 initiates the feed forward activation of EZH2, which in turn represses miR-31. Analysis of the SOX4 3′-UTR using microrna.org (maintained at cBio, the Computational Biology Center at Memorial Sloan-Kettering Cancer Center) predicted a miR-31 binding site (Figure 4A). A sequence alignment search showed that the miR-31 target sequence in the SOX4 3′-UTR is conserved in humans and most great apes (Figure 4A). To test whether SOX4 is regulated by miR-31 through direct binding to its 3′UTR, we used psiCHECK2 SOX4 full length 3′-UTR plasmid (WT) [3], and constructed two derivatives, SOX4 WT 3′-UTR oligo plasmid (WT OLIGO) and SOX4 mutant 3′-UTR oligo plasmid (MUT OLIGO) (Figure 4A). The WT OLIGO plasmid contained a 71-nucleotide region including the miR-31 target sequence. In the SOX4 mutant 3′-UTR (MUT OLIGO), 4 nucleotides in the seed sequence were mutated [3]. When co-transfected into HEK-293 cells, the luciferase reporter, SOX4 WT 3′-UTR and miR-31 plasmid showed reduced luciferase activity compared to co-transfection with miR-31 empty control vector (Figure 4B). This suppressive effect was reversed by the four-nucleotide substitution in the miR-31 binding sequence. Similarly, the suppressive effect of miR-31 on the SOX4 3′-UTR activity was observed in the esophageal tumor cell lines, TE8 and FLO1 (Figure 4B). In line with these results, overexpression of miR-31 in FLO1 cells suppressed the expression of SOX4 at both the protein (Figure 4C) and mRNA level (Figure 4D). As previously reported [11], our data confirm that miR-31 inhibits EZH2 expression (Figure 4C and D) whereas EZH1 expression was unchanged. Interestingly, miR-31 decreased HDAC3 on protein (Figure 4C) and mRNA levels (Figure 4D). However, target prediction algorithms do not detect any putative binding site for miR-31 in the 5′UTR, 3′UTR or coding sequence of HDAC3. Taken together, these results demonstrate that SOX4 is a direct target of miR-31, while EZH2 and HDAC3 are indirect targets.

To analyze the regulation of miR-31, EZH2, EZH1 and HDAC3 by SOX4, we chose TE11 cells, which express low SOX4 and EZH2 levels, had high expression of miR-31 as a model. Ectopic expression of SOX4 in TE11 cells (Figure 4E) decreased miR-31 expression, although this was not statistically significant (Figure 4F). Additionally, we observed that SOX4 induced EZH2 expression (Figure 4F) as previously reported [30], but not EZH1. HDAC3 expression increased as well. Consistent with this observation, overexpression of EZH2 in TE11 cells (Figure 4G) led to a significant decrease in miR-31 expression (Figure 4H). Interestingly, EZH2 induced SOX4 expression by more than a 2-fold (Figure 4H), suggesting that not only does SOX4 regulate EZH2, but EZH2 also regulates SOX4 thereby potentially repressing miR-31 and/or other miRNAs and transcription factors.

### Depletion of SOX4 suppresses growth, migration and invasion of esophageal cancer cells

**Figure 5:**
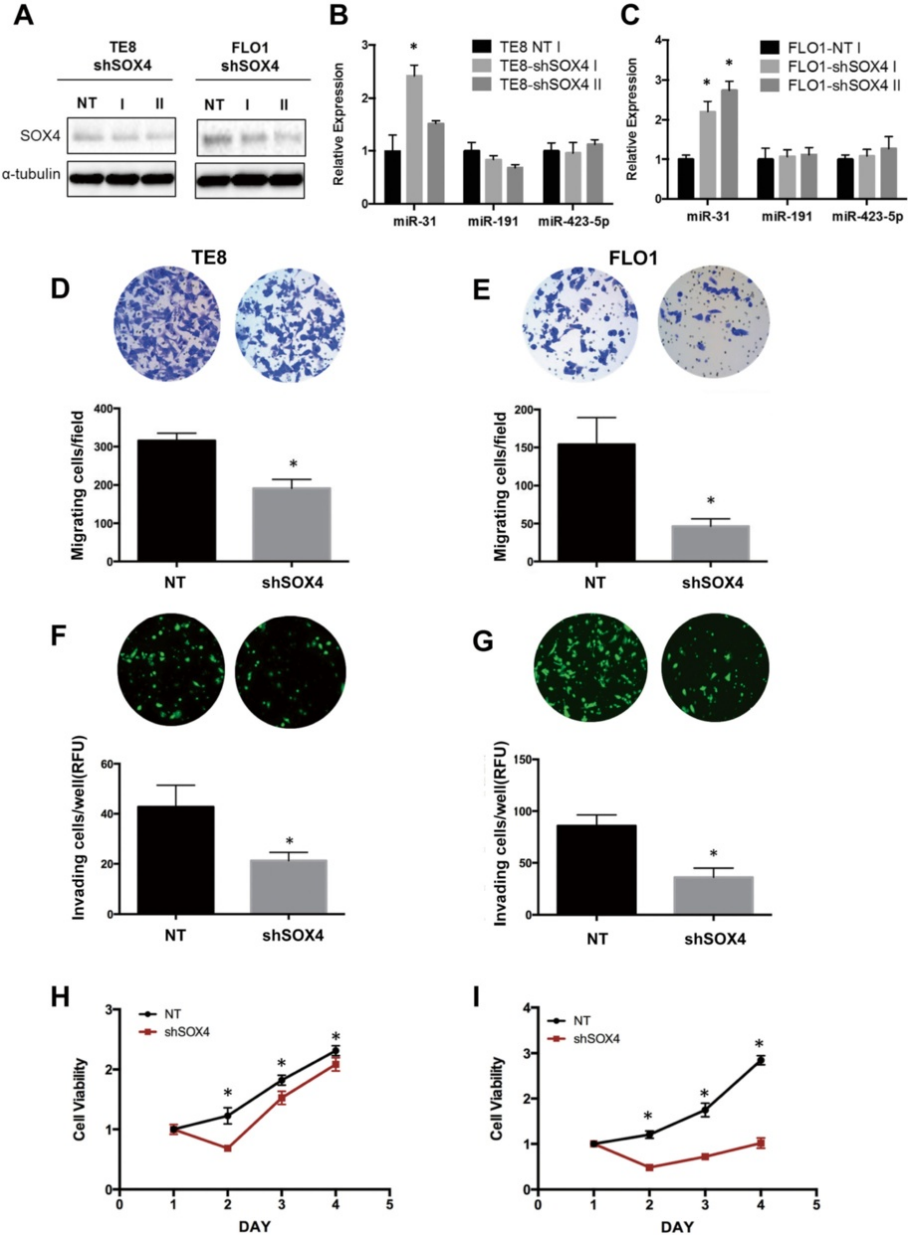
**SOX4 knockdown suppresses migration, invasion and growth of invasive esophageal cancer cells.** TE8 and FLO1 cells were transfected with non-targeting shRNA control or shSOX4. **(A)** SOX4 protein expression was analyzed by Western blotting. **(B, C)** miR-31 expression in response to SOX4 knockdown was measured by qRT-PCR, miR-191 and miR-423-5p were used as controls. miR-31, miR-191, miR-423-5p expression was normalized to RNU6. **(D, E)** Cell migration was measured using Boyden chamber transwell assays 24 hour post-transfection. **(F, G)** Invasion was measured by Marigel-coated transwell assays 24 hour post-transfection. **(H, I)** Cell viability was evaluated using the WST-1 assay. Results are means ± SD from at least three biological replicates.

Based on the observation that miR-31 targets both SOX4 and EZH2 and given that SOX4 binds to the EZH2 promoter to activate its transcription, we examined whether SOX4 leads to feed forward activation of EZH2, and subsequent miR-31 silencing. TE8 and FLO1 cells, which express high levels of SOX4 and EZH2, showed the lowest expression level of miR-31. SOX4 knockdown (Figure 5A) using shRNA led to significant upregulation of miR-31 in TE8 and FLO1 cells, while miR-191 and miR-423-5p, used as controls, did not show any significant change (Figure 5B and C). We next investigated if loss of SOX4 functionally mimics overexpression of miR-31 in esophageal cancer cells. To assess the role of SOX4, we studied the migratory and invasive potential of TE8 and FLO1 cells following SOX4 knockdown with shRNA. Suppression of SOX4 inhibited transwell migration (Figure 5D and E) and invasion through Matrigel-coated Boyden chambers (Figure 5F and G) in TE8 and FLO1 cells. SOX4 knockdown cells also showed a significant reduction in proliferation (Figure 5H and I). Taken together, these data implicate SOX4 as a key mediator of the tumor-suppressive effects of miR-31 in this system.

### SOX4, HDAC3 and EZH2 form a co-repressor complex to inhibit miR-31 expression

**Figure 6:**
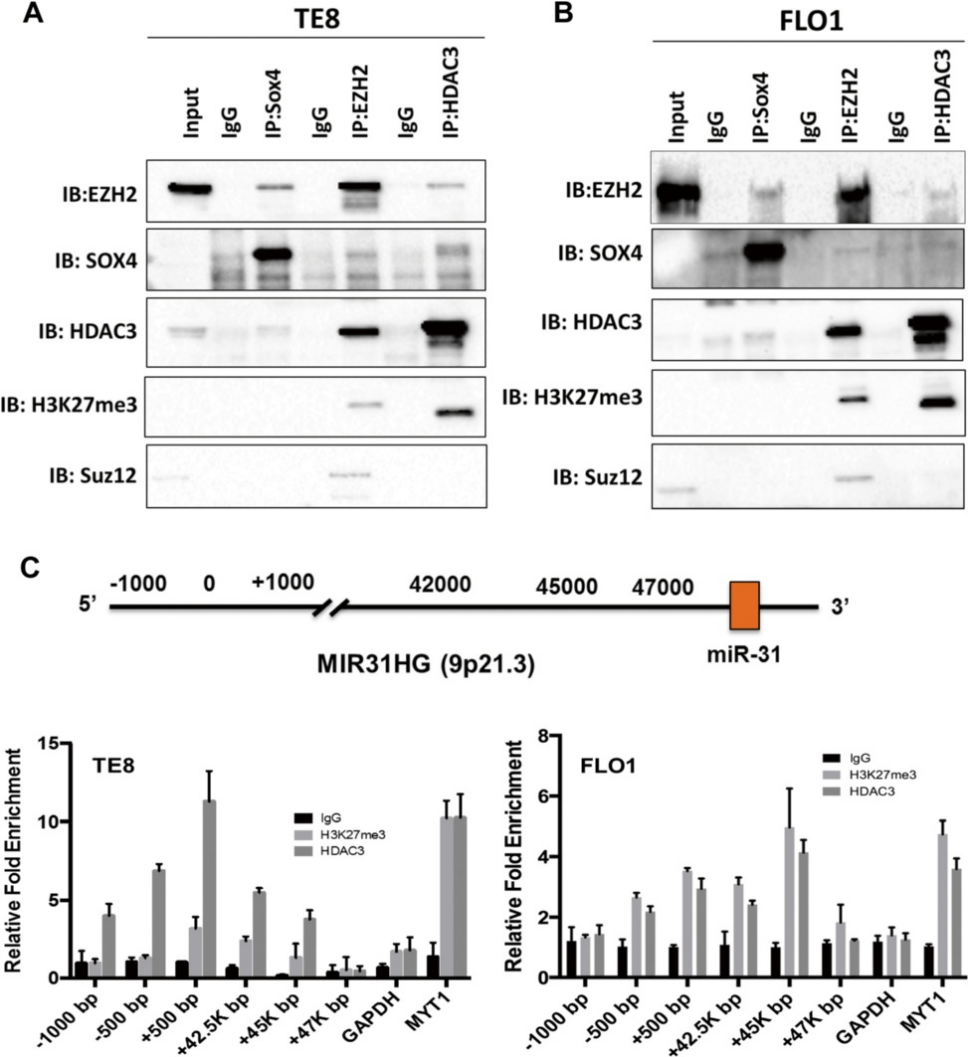
**SOX4 interacts with EZH2 and HDAC3. (A)** TE8 cell lysates were immunoprecipitated with SOX4, EZH2, HDAC3 or IgG control antibodies and immunoblots were probed for indicated antibodies. **(B)** FLO1 cell lysates were pulled-down with SOX4, EZH2, HDAC3 antibody or IgG control and immunoblots were probed for indicated antibodies. **(C)** Quantitative ChIP assay showing H3K27me3, and HDAC3 enrichment on the proximal and distal promoters regions of miR-31 and its host genes MIR31HG.

PRC2 is known to recruit DNA methyl-transferases (DNMTs), HDACs and other chromatin modifying enzymes to repress the transcription of developmental genes [43]. Because SOX4 was recently shown to interact with other transcription factors, we tested whether SOX4 forms a co-repressor complex with EZH2 and HDAC3 to silence miR-31 expression. Using anti-SOX4 antibody for co-immunoprecipitation, cell lysates from TE8 (Figure 6A) and FLO1 cells (Figure 6B) showed interactions between SOX4 and EZH2, HDAC3 and H3K27me3 (Figure 6A, B). We next performed co-immunoprecipitation assays with anti-EZH2 antibody and showed that EZH2 equally interacts with SOX4 (Figure 6A, B). Immunoprecipitates obtained with EZH2 antibody detected also HDAC3, H3K27me3, and Suz12 (Figure 6A, B). To test whether HDAC3 interacts with SOX4 and EZH2, HDAC3-antibody was used for pull down. Lysates immunoprecipitated with HDAC3-specific antibody contained EZH2, SOX4, H3K27me3 in TE8 cells (Figure 6A), but only a faint band of EZH2 in FLO1 cells (Figure 6B). Suz12 was not detected after pull-down with HDAC3-specific antibody in either cell line.

Finally, we tested whether SOX4, EZH2 and HDAC3 tethered to the miR-31 promoter. Previous reports identify CpG islands and histone trimethylation mark sites upstream of the miR-31 promoter [8]. Therefore, we tested whether SOX4, EZH2 and HDAC3 bind to those regions. ChIP analysis denoted H3K27me3 and HDAC3 enrichment in regions upstream of miR-31 (Figure 6C). However, we were unable to detect SOX4 at miR-31 promoter regions (data not shown). HDAC3 and H3K27me3 were equally enriched at the MYT1 promoter, which was used as a positive control as EZH2 has been shown to bind and regulate MYT1 promoter activity [44]. We conclude that SOX4, HDAC3 and EZH2 function as a potential co-repressor complex to silence miR-31.

### SOX4, EZH2 and HDAC3 inversely correlate with miR-31 expression in invasive esophageal cancer cells

**Figure 7:**
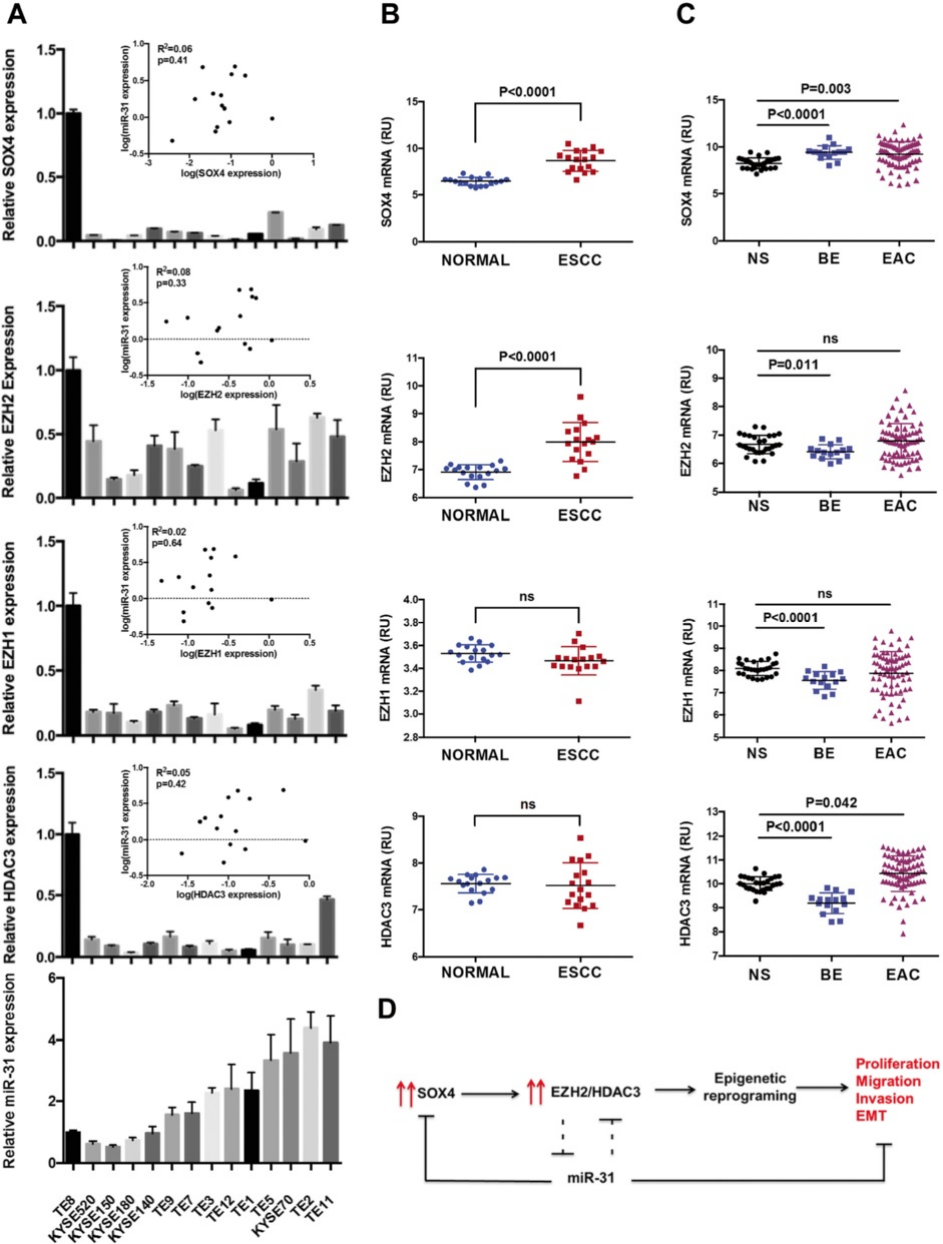
**SOX4, EZH2 and HDAC3 are upregulated and inversely correlate with miR-31 in esophageal cancers. (A)** qRT-PCR of SOX4, EZH2, EZH1. HDAC3 and miR-31 in 16 ESCC cell lines. Insert: correlation between mRNA expression of SOX4, EZH2, EZH1, HDAC3 and miR-31 in ESCC cell lines. Statistical analysis was done by ANOVA. **(B)** SOX4, EZH2, EZH1 and HDAC3 expression in primary esophageal squamous cell carcinomas, ESCC, compared to adjacent normal tissues (Geo dataset GSE20347). **(C)** SOX4, EZH2, EZH1 and HDAC3 expression in Barrett’s Esophagus lesions and primary esophageal adenocarcinoma tumors, EAC, compared to adjacent normal tissues (Geo dataset GSE13898). **(D)** Model depicting reciprocal inhibition of miR-31 and SOX4, EZH2 and HDAC3.

To determine the expression of the complex-forming partners, SOX4, EZH2 and HDAC3, in esophageal cancer cell lines and tissues, we used qRT-PCR. We profiled SOX4, EZH2 and HDAC3 in ESCC (Figure 7A) and EAC (data not shown) cell lines and found a strong, inverse correlation between miR-31 and expression of SOX4, EZH2 and HDAC3 in invasive cancers of both histologies. Probing various publicly available datasets (GSE20347, GSE47404, GSE13937) to assess the expression of SOX4, EZH2 and HDAC3 in primary esophageal squamous and adenocarcinoma samples, we found SOX4 and EZH2 to be significantly upregulated in ESCC tumor samples (GSE20347), compared to normal tissues (Figure 7B). In another ESCC dataset (GSE47404), only EZH2 expression (*P value = 0.008*) was significantly upregulated in metastatic compared to non-metastatic patients. In EAC dataset (GSE13937), we found that SOX4 (*P value = 0.003*) and HDAC3 (*P value = 0.042*) were increased in tumor samples compared to adjacent normal tissues (Figure 7C). Taken together, these data identify a molecular circuit where SOX4, EZH2 and HDAC3 target miR-31 to promote esophageal malignancy. Inversely, in non-invasive tumor cells miR-31 targets SOX4, EZH2 and HDAC3 by direct and indirect means to inhibit tumor cell invasion (Figure 7D).

## Discussion

Differential microRNAs expression has been linked to tumor initiation and progression. Depending on cellular context, microRNA expression in tumors may be increased or decreased, with microRNAs behaving as tumor promoters or suppressors, respectively [2]. Moreover, multiple microRNAs have been shown to promote or inhibit metastasis [3,45]. Because metastasis is responsible for more than 90% of cancers-related deaths, it is important to define molecular mechanisms by which microRNAs regulate metastasis and define new therapeutic targets. Studies on the role of miR-31 in esophageal tumors have produced conflicting results [16,18,19,46]. Hence, it is important to define not only the molecular pathways regulated by miR-31 but also the factors regulating miR-31 expression and functions in various tissues and cancers. The lack of consistency in the literature with respect to miR-31 expression in esophageal cancer may be due to platform choice or normalization methods. Variability may also result due to the limited number of patients in each study. Here, we show downregulation of miR-31 in invasive and aggressive esophageal cancer cells. miR-31 has a known role in prostate cancer and melanoma, suppressing key cell cycle regulators and pro-oncogenic genes such as CDK1, E2F2, EXO1, FOXM1, MCM2, Src or MET [11]. We show that miR-31 significantly suppresses migration and invasion *in vitro* in both aggressive esophageal adenocarcinoma and squamous cell carcinoma. Ectopic expression of miR-31 did not significantly affect tumor cell proliferation, causing only a marginal decrease in colony formation in the adenocarcinoma cell line FLO1. Interestingly, Valastyan *et al.* also reported that miR-31 promoted metastasis but not cell proliferation in breast cancer [9]. A number of studies argue that epithelial-mesenchymal transition (EMT) plays a crucial role in cancer metastasis and progression, and loss of miR-200 family members drives EMT in multiple cancers [47,48]. We examined expression of EMT markers in miR-31-overexpressing cell lines but found no significant alterations (data not shown). We conclude that miR-31 is not a strong inhibitor of EMT.

Several mechanisms could contribute to aberrant miR-31 expression in cancer. Based on data demonstrating epigenetic repression of miR-31 expression by DNA methylation and EZH2-mediated H3K27me3 epigenetic mark in melanoma, leukemia and prostate cancer [8,11], we examined the role of epigenetic regulation of miR-31 in invasive esophageal cancer. Our results indicate that DNA methylation and Polycomb-mediated histone methylation both contribute to miR-31 silencing, since treatment with the EZH2 inhibitor, DZNep, and the DNMT inhibitor, AZA, enhanced miR-31 expression. Additionally, we show for the first time that histone deacetylation contributes to miR-31 silencing as treatment with the pan-HDAC inhibitor SAHA restored miR-31 expression. This is in line with recent observations that EZH2 interacts with HDAC3 to downregulate miR-29 [37] and that EZH2 can interact with DNMT to control DNA methylation [49]. Therefore, we propose a key role for Polycomb/EZH2, HDAC and DNMT for survival and metastasis of esophageal cancers.

Recent work argues that SOX4 is a master regulator of EMT and cell invasion through its binding to the EZH2 promoter, inducing EZH2 transcription [30]. Our results indicate that miR-31 down-regulates SOX4 by binding to its 3′-UTR in EAC and ESCC cells. Consistent with previous reports [8,11], we found that miR-31 negatively regulates EZH2 as well. Furthermore, we observed a significant decrease in HDAC3 expression with miR-31 overexpression. We, therefore, propose that one underlying mechanism by which miR-31 suppresses tumor cell invasion is by directly targeting SOX4 and indirectly targeting EZH2 and HDAC3. Notably, overexpression of SOX4 is observed in a variety of human cancers [21]. Loss of SOX4 led to a significant increase in miR-31 expression and strongly inhibited tumor cells proliferation, migration and invasion. Additional studies will aim to identify a subset of patients with concomitant high SOX4, EZH2 and HDAC3 and low miR-31 to demonstrate the mechanistic and clinical correlation between these pathways.

Multiple reports show that the polycomb PRC2/EZH2 interacts with HDAC to repress the transcription of CDH1 [38]. Moreover, MYC was recently shown to interact with EZH2 and HDAC3 to repress miR-29 in lymphomas [37]. However, it is not clear how PRC2/EZH2 is recruited to the promoter of specific genes. Similarly, SOX4 has been shown to interact with multiple transcription factors. Despite the fact that our computational analysis did not detect any SOX4 regulatory elements upstream of the miR-31 promoter, co-IP assays show that SOX4 interacts with EZH2 and HDAC3. Based on the ChIP experiments, we propose that EZH2 and HDAC3 bind to a similar region on the miR-31 promoter, confirming that histone methylation and histone deacetylation contribute to miR-31 repression. It is also possible that other molecules such as PRC2-interacting YY1 or the SOX4-interracting protein GATA4 mediate SOX4 recruitment to the DNA. Future studies are needed to confirm such possibilities.

## Conclusions

Taken together, our data identify a molecular circuit where SOX4, EZH2 and HDAC3 cooperate to repress miR-31. This may lead to tumor progression and the metastasis of EAC and ESCC; SOX4, EZH2, HDAC3 and miR-31 emerge as potential therapeutic targets.

## Materials and methods

### Cell lines

The squamous cell carcinoma cell lines TE8, TE1, TE11, TE7, TE12, TE2, TE3, TE5 and TE9 were developed and characterized by Kuroki et al. [50]. OE33 and FLO1 were established by Dr. David Beer (University of Michigan). HEK293T, CP-A and CP-B were purchased from American Type Culture Collection (ATCC). KYSE140, KYSE180, KYSE150, KYSE520, KYSE70 were developed and characterized by Shimada et al. [51]. All cells were cultured at 37°C in 5% CO_2_.

### Chemicals and antibodies

Suberoyl anilide hydroxamic acid (SAHA, a pan-HDAC inhibitor) and 3-Deazaneplanocin A (DZNep, a polycomb EZH2 subunit inhibitor) were purchased from Millipore, Temecula, CA. The DNA methylation inhibitor 5-AZA-Cytidine (AZA) was from Sigma, St. Louis, MO. Cells were treated with indicated concentration and incubated for 72 hours before harvesting. Agarose A/G plus is from Santa Cruz. The following antibodies were used as primary antibodies: SOX4 (Santa Cruz; 1:1000); EZH2 (BD; 1:4000); HDAC3 (Millipore; 1:4000); total histone H3 (Abcam; 1:5000); Tri-methylated histone H3 H3K27me3 (Millipore; 1:3000); SUZ12 (Santa Cruz; 1:1,000); EZH1 (Santa Cruz; 1:1,000); β-tubulin (Sigma-Aldrich; 1:5,000).

### Oligonucleotides and plasmids

The pBABE-miR-31 plasmid (Plasmid#26088), pWPXL-SOX4 (plasmid#36984), pCMVHA-hEZH2 (plasmid#24230), Psicheck2 SOX4 full-length 3′UTR (Plasmid#26989) were purchased from Addgene (Cambridge, MA).

A 71 bp WT fragment of the SOX4 3′UTR (SOX4 WT OLIGO) was created by overlapping extension PCR and cloned between the XhoI and NotI site of the psicheck2 plasmid. Similarly, the mutant construct of SOX4 3′UTR (SOX4 Mutant OLIGO) which carried a substitution of four nucleotides within the core seed sequence of miR-31, was carried out using overlapping extension PCR and cloned between the XhoI and NotI site of the psicheck2 plasmid.

The two set of plasmids containing shRNA specific to SOX4 and EZH2 were purchased from OriGene (Rockville, MD).

Primers used for SOX4, forward: 5′-AGCGACAAGATCCCTTTCATTC-3′, reverse: 5′-CGTTGCCGGACTTCACCTT-3′; EZH2, forward: 5′-GTACACGGGGATAGAGAATGTGG-3′, reverse: 5′GGTGGGCGGCTTTCTTTATCA-3′, for EZH1, forward: 5′-ATGCGACTTCGACAACTTAAACG-3′, reverse: 5′-GGCTTCATTGACTGAACAGGTT-3′, HDAC3, forward: 5′-CCTGGCATTGACCCATAGCC-3′, reverse: 5′-CTCTTGGTGAAGCCTTGCATA-3′, GAPDH, forward: 5′-GCGACACCCACTCCTCCAC-3′, reverse: 5′-TCCACCACCCTGTTGCTGTAG-3′.

### 3′UTR Luciferase Reporter assays

HEK293, TE8 and FLO1 cells were plated in triplicate in a 24 well plate. One day after plating, cells were transfected with the dual Renilla and Firefly luciferase reporter plasmid (psiCHECK-2) containing the full length 3′UTR of SOX4 (plasmid# 26989), the short WT oligo or mutant oligo along with a pBABE-miR-31 or pBABE Empty Vector expressing plasmid using FuGene HD (Promega). 48 hr post-transfection, cells were lysed using 1X passive lysis buffer and lysates were analyzed using the Dual-Glo Luciferase Reporter Assay System (Promega) on the Synergy4 multi-mode microplate reader (BioTeK).

### Quantitative real time PCR

Total RNA was isolated from cells with Qiazol reagent (Qiagen) and reverse transcribed into cDNA using the miRNeasy mini kit and miScript miRNA Reverse Transcription kit (Qiagen). qRT-PCR was performed according to the manufacturer’s instructions (Applied Biosystems).

### Proliferation assay

WST-1 reagent (Roche) was used according to manufacturers’ protocols to assess cell viability.

### Cell migration and invasion assays

Migration and Invasion assays were performed as previously described [52]. Migrating or invading cells were then photographed using the Zeiss Axioskop Plus or eVos microscope and quantified with ImageJ software.

### Colony formation assay

For clonogenic assay, 500 transfected cells were seeded in six-well plates and maintained in complete medium for 2 weeks. Colonies were fixed with ice-cold methanol and stained with crystal violet. Colonies were photographed and counted using GelCount (Oxford Optronix).

### Western blot analysis

Western blot was performed as previously described [52].

### Immunoprecipitation (IP)

Cells were collected and lysed in IP lysis buffer (150 mM NaCL, 50 mM Tris pH8, 1% Triton X-100, 1% NP-40) supplemented with protease and phosphatase inhibitors, incubated on ice for 20 min, and cleared by centrifugation at 13,200 rpm at 4 C for 20 min. Total protein lysate (500 μg) was immunoprecipitated with the agarose-immobilized antibody (6 μg of anti-SOX4, EZH2, HDAC3 or isotype control antibodies) and incubated overnight at 4°C. Immune complexes were eluted from the agarose beads and analyzed by SDS-PAGE followed by immunoblot analysis. For co-IP in 293 T, cells were transfected with plasmids using FuGene HD (Promega). Cells were collected 48 hours post-transfection and analyzed as described above.

### Chromatin Immunoprecipitation (ChIP)

The ChIP assays were adapted and performed according to previous publications [53]. Briefly, cells were fixed using 1% formaldehyde for 15 minutes and quenched using 125 mM glycine for 5 minutes at room temperature. After centrifugation the cell pellet was re-suspended in the cell lysis buffer (150 mM NaCL, 50 mM Tris pH8, 1% Triton X-100, 1% NP-40, 0.01% SDS, 1.2 mM EDTA pH 8.0, 1 mM PMSF). Protein-bound chromatin was fragmented by sonication. Equal volumes of chromatin were immunoprecipitated with anti-HDAC3, anti-SOX4, anti-EZH2, anti-trimethyl-Histone H3 Lys27 or normal IgG as a negative control (Millipore). Following extensive washing the immunoprecipitated DNA was treated with RNase (Qiagen) for 30 min at 37°C and proteinase K (Roche) for an hour at 45°C. The DNA was eluted using 100 mM NaHCO3 and 1% SDS and the crosslinks were reversed using 300 mM NaCl at 65°C for 16 hours. Immunoprecipitated DNA and whole cell extract DNA were purified by Qiaquick PCR purification kit (Qiagen). The purified DNA was amplified by real-time quantitative PCR with Qiagen QuantiTech SYBR Green PCR master mix and analyzed for enrichment. Real-time qPCR amplification was performed with Applied Biosystems StepOnePlus real time PCR system.

Primers used as described in Lin et al. [44]: -1,000bp forward: CCGATGACCTAGCCAGAAGT, reverse: CCCCACCCTTCAACTCGTAG; -500 bp, forward: TATCCTCAACCCTCCGTGTC, reverse: CATACACCTGAAGGGGCAGT; +500 bp, forward: CAATTTTGGCCCAGGAGATA, reverse: TTTCCGGGGACCTCTAGTTT; +42,500 bp, forward: TGGCCTATTTGCTGTTCTAATGAC, reverse: GCAAGCCAACCCCAACA; +45,000 bp, forward: AATGGGCCCTGCATTCTCT, reverse: AAAACCCACACCCTCACCAC; +47,500 bp, forward: CATCTTCAAAAGCGGACACTCT, reverse: ACAATACATAGCAGGACAGGAAG; MYT, forward: AGGCACCTTCTGTTGGCCGA, reverse: AGGCAGCTGCCTCCCGTACA; GAPDH, forward: CGGCTACTAGCGGTTTTACG, reverse: AAGAAGATGCGGCTGACTGT.

### Dataset analysis

Datasets made publicly available from GEO Datasets (http://www.ncbi.nlm.nih.gov/gds/). The collected information from each dataset was analyzed and visualized in Prism version 6.00 for Mac (GraphPad software, La Jolla, California).

### Statistical analysis

Each experiment was repeated at least three times. Numerical data are presented as mean ± standard deviation. The differences between two groups were analyzed using a Student’s t-test (two-tailed) or two-way ANOVA. Differences were considered statistically significant at p < 0.05. All statistical analysis was performed on GraphPad Prism 6.0c software (La Jolla, CA).

## Abbreviations

ESCC: Esophageal squamous cell carcinoma
EAC: Esophageal adenocarcinoma
HNSCC: Head and neck squamous cell carcinoma
3′UTR: 3′Untranslated region
WT: Wild type
AZA: 5-Azacytidine
DZNep: 3-Deazaneplanocin
SAHA: Suberoyl anilide hydroxamic acid
